# The Knowledge, Attitude, and Perception (KAP) of Healthcare Professionals in Pediatric Settings Toward Oral Manifestations of Inflammatory Bowel Disease (IBD): A Survey-Based Cross-Sectional Study

**DOI:** 10.3390/jcm15041598

**Published:** 2026-02-19

**Authors:** Stefania Leuci, Roberta Benvenuto, Gennaro Musella, Simone Liguori, Gaetano Marenzi, Francesco Riccitiello, Michele Davide Mignogna

**Affiliations:** 1 Department of Neuroscience, Reproductive and Odontostomatological Sciences, University of Naples Federico II, 80131 Naples, Italy; stefania.leuci@unina.it (S.L.); rob.benvenuto@studenti.unina.it (R.B.); gaetano.marenzi@unina.it (G.M.); francesco.riccitiello@unina.it (F.R.); mignogna@unina.it (M.D.M.); 2 Department of Clinical and Experimental Medicine, University of Foggia, 71122 Foggia, Italy; gennaro.musella@unifg.it

**Keywords:** KAP, IBD, Crohn’s disease, ulcerative colitis, early-onset, pediatric, oral, education

## Abstract

**Objectives**: This study aimed to evaluate the knowledge, attitudes, and perceptions of Italian general dentists, pediatric dentistry residents and pediatric residents regarding IBD-related oral manifestations, in order to identify educational gaps and promote a multidisciplinary approach. **Methods**: A cross-sectional survey using a validated questionnaire was conducted among pediatric residents, pediatric dentistry residents and general dentists. The tool included sociodemographic questions, 30 true/false items on knowledge and 20 Likert-scale items on attitude and perception. Data were collected online and on paper and analyzed using descriptive statistics, chi-square tests, and ANOVA. **Results**: Out of 228 respondents, general knowledge of IBD was high, while specific knowledge about oral manifestations was limited. Pediatric dentistry residents and pediatric residents performed significantly better than general dentists on targeted items (*p* = 0.01). Attitudinal responses revealed low clinical confidence, with only a minority feeling prepared to recognize or manage oral lesions, though most were willing to pursue further education. Perception was overall positive, with strong support for multidisciplinary collaboration (96.5%), and 89.5% recognized the role of dentists in early IBD detection. General dentists more often reported the need for additional training (*p* = 0.02). No significant differences emerged by sex or age. **Conclusions**: Our study highlights significant knowledge gaps and limited clinical confidence but also reveals a strong willingness to improve and collaborate. While the number of children with IBD seen by general dentists and primary care pediatricians is limited, considering the increasing incidence of pediatric IBD, our results support the need for targeted educational interventions.

## 1. Introduction

Chronic inflammatory bowel diseases (IBDs) represent a group of gastrointestinal disorders characterized by persistent inflammation of the digestive tract. The two primary forms are Crohn’s disease (CD) and ulcerative colitis (UC), which differ in terms of anatomical distribution, histopathological features, and clinical course. CD may involve any part of the gastrointestinal tract [1] and is characterized by transmural inflammation affecting all layers of the bowel wall, including the deeper tissues. A distinguishing feature of CD is the presence of “skip lesions”, discontinuous areas of inflammation, along with a propensity to develop complications such as deep ulcerations, strictures, fistulas, abscesses, and bowel obstruction [2]. In contrast, UC predominantly affects the colon and rectum, with inflammation confined to the mucosal layer of the intestinal wall [3]. Unlike CD, UC lesions are typically continuous and limited to superficial layers and do not exhibit the penetrating or stricturing phenotypes associated with CD. IBD approximately affects 5 million individuals worldwide and 100,000 to 200,000 individuals in Italy. Its prevalence is highest in industrialized countries but is rising considerably in developing regions [4,5,6]. Although the precise etiology remains uncertain, the prevailing hypothesis suggests that IBD results from an inappropriate immune response to intestinal microbiota in genetically susceptible individuals, influenced by environmental and immunological factors [4,7,8,9,10]. IBD commonly manifests in young adulthood, with a peak incidence between 15 and 30 years of age; however, pediatric cases have been increasingly reported, and an incidence of 21 and 3.7 per 100,000 per year for early-onset (ages 6–17) and very early-onset (before 6 years) IBD, respectively, has been reached in the most affected areas [11,12,13]. Beyond gastrointestinal symptoms, IBD is frequently associated with extraintestinal manifestations (EIMs), which may arise as direct or indirect consequences of chronic intestinal inflammation [14]. EIMs can affect multiple organ systems, most commonly the joints, skin, eyes, hepatobiliary tract, and oral cavity [15,16]. The oral mucosa represents a frequent site of extraintestinal involvement, especially in patients with Crohn’s disease, where oral manifestations are reported in 20–50% of cases, compared with approximately 8% in ulcerative colitis [17,18].

Oral lesions in IBD may be classified as specific or non-specific based on the presence of granulomas in histopathological examination [19]. Among the specific lesions, lip swelling is one of the most common and typically presents as diffuse, symmetric or asymmetric enlargement of one or both lips, with a slight predilection for the lower lip; the swelling may persist for weeks or months. Another characteristic finding is cobblestoning of the oral mucosa, resulting from mucosal ridging and hyperplasia that produce a nodular appearance with interspersed fissures, resembling a cobblestone pavement [15,20]. Additionally, tag-like lesions—small, pale, or whitish fibrous protuberances due to epithelial hyperplasia—are frequently observed in the labial and buccal vestibules or the retromolar area [21]. Non-specific lesions are more prevalent and include aphthous stomatitis and pyostomatitis vegetans (PV). Aphthous ulcers, which occur in up to 10% of UC patients and 20–30% of those with CD [22], present as round, shallow erosions with a central fibrinous membrane surrounded by an erythematous halo. PV is a rare, benign, chronic inflammatory mucocutaneous condition characterized by thickened, erythematous oral mucosa with multiple non-removable pustules and superficial erosions.

Early diagnosis of IBD is essential for timely therapeutic intervention, prevention of complications, and improved quality of life. Oral lesions, which may precede gastrointestinal symptoms by several years [19,23,24], can serve as early diagnostic clues—an especially pertinent consideration in pediatric populations, where these manifestations are more common [25,26]. Therefore, the recognition of oral EIMs by dental professionals, particularly pediatric dentists, may facilitate prompt referral and diagnosis, potentially altering disease trajectory. Dentists should be equipped not only to identify these manifestations but also to manage and monitor affected patients, especially in light of the link between IBD, bacterial dysbiosis and systemic inflammation, which underscores the importance of periodontal health [27]. In this context, dentists become essential members of the multidisciplinary care team, collaborating with gastroenterologists and other healthcare professionals to deliver comprehensive, patient-centered care. Most existing studies in the literature primarily concentrate on clinical aspects such as the epidemiology, diagnosis, and treatment of oral lesions in these patients, without systematically exploring healthcare professionals’ knowledge, attitude, and perception [15,28,29,30,31].

Furthermore, there is a lack of studies investigating how training and professional experience influence the attitudes and perceptions of physicians and dentists toward managing pediatric patients with oral manifestations of IBD.

Therefore, the aim of this study was to evaluate the knowledge, attitude and perception (KAP) of Italian general dentists, pediatric dentistry residents and pediatric residents regarding oral lesions in patients with IBD, aiming to identify educational strategies that could improve the quality of care for these patients.

## 2. Materials and Methods

### 2.1. Study Description

This study was conducted by the Oral Medicine Unit, Department of Neurosciences and Reproductive and Odontostomatological Sciences, University of “Federico II,” Naples, Italy. The study complies with the ethical principles of the World Medical Association’s Declaration of Helsinki, and the methods employed were approved by the Ethics Committee of the University of Naples Federico II (No. 437/20).

Participants included three groups: general dentists, pediatric dentistry residents, and pediatric residents. Inclusion criteria were as follows: for the first group, participants were required to hold a degree in dentistry without any postgraduate specialization and be registered with the Italian medical and dental council; for the second group, participants were required to be enrolled in a postgraduate school of pediatric dentistry; for the third group, participants were required to be enrolled in a postgraduate school of pediatrics. All participants were required to be fluent in Italian to complete the questionnaire thoroughly.

### 2.2. Questionnaire Design

The study used questionnaire was divided into four sections:

1. Demographic data: this section collected demographic information, including sex, age, profession (general dentist/pediatric dentistry resident/pediatric resident), year and university of degree completion, and geographic region of practice.
2. Knowledge: this section included 30 items regarding oral lesions in patients with IBD, aimed at assessing participants’ knowledge on the topic. Each item was answered dichotomously (true/false). Correct responses were scored +1 and incorrect responses 0. The percentage of correct answers was calculated for each participant (Table 1).
3. Attitude: this section comprised 10 items to evaluate participants’ attitudes toward managing oral lesions in patients with IBD. Items were rated on a 5-point Likert scale: (1) Strongly disagree, (2) Disagree, (3) Neither agree nor disagree, (4) Agree, and (5) Strongly agree (Table 2).
4. Perception: this section included 10 items to assess participants’ perceptions regarding the management of oral lesions in patients with IBD, rated on the same 5-point Likert scale as above (Table 3).

No validated instrument specifically designed to assess knowledge, attitude, and perception regarding oral manifestations of IBD among general dentists, pediatric dentists, and pediatricians was available in the literature. Consequently, the questionnaire was developed based on a non-systematic review of the scientific literature, which included English-language articles published in journals indexed in Web of Science up to 2 December 2024. The literature search was conducted using major databases, PubMed, Embase, Web of Science, and Scopus, employing specific key words (MeSH terms and EMTREE thesauri) related to oral lesions in IBD. Among the terms used were: oral manifestation; inflammatory bowel disease; and pediatrics. Main topics related to the epidemiology and clinical features of oral lesions in patients with IBD were identified and subsequently translated into questionnaire items. The Italian and English versions of the questionnaire, along with the scientific articles consulted for the item formulation, are provided in the Supplementary Materials.

During the questionnaire design process, particular attention was paid to synthesizing the information into clear and precise questions, ensuring they were easily understandable by general dentists, pediatric dentistry residents and pediatric residents while maintaining technically accurate yet accessible language. The final questionnaire was organized into thematic sections, each focusing on a specific component of the KAP framework.

### 2.3. Translation and Validation

The questionnaire, originally drafted in English, was translated into Italian following a rigorous blind backward translation process, in accordance with the methodology described by Sousa & Rojjanasrirat [32]. Two bilingual researchers conducted the forward and backward translation procedures to ensure both linguistic and cultural equivalence. The final Italian version was reviewed by a multidisciplinary committee, comprising the same translators and domain experts, to confirm the content validity.

Subsequently, the questionnaire was administered to a pilot group representative of the target population. Each participant was asked to assess the clarity of the instructions and the scale items using a dichotomous scale (clear or unclear).

### 2.4. Data Collection

Data collection was carried out using two modes of questionnaire administration: paper-based and online. A portion of the participants were invited to take part in the study through paper questionnaires distributed at the Schools of Medicine and Dentistry or in private dental practices by a trained research team member. For the remaining participants, the questionnaire was distributed via email, and they were invited to complete it independently using Google Forms. The Google Forms questionnaire was configured to require mandatory responses for all items. Participation was voluntary and all responses were anonymous. Before completing the questionnaire, participants were informed about the purpose of the study, the voluntary nature of participation and the anonymous processing of data; informed consent was obtained from all respondents. The average completion time was approximately 15 min. Paper questionnaires were subsequently digitized for data processing.

### 2.5. Reliability and Validity

To assess test–retest reliability, the questionnaire was administered twice to a subgroup of 20 participants, with a three-week interval between the two sessions. The intraclass correlation coefficient (ICC) was calculated with a 95% confidence interval using a two-way mixed-effects model for the mean of the ratings and absolute agreement.

For all statistical tests, a *p*-value of less than 0.05 was considered statistically significant. In cases of multiple comparisons, *p*-values were adjusted using Bonferroni correction.

### 2.6. Statistical Analysis

Statistical analyses were performed using IBM SPSS Statistics version 29.0, IBM Corp., Armonk, NY, USA. Categorical variables were described using absolute and relative frequencies. Continuous variables, such as knowledge, attitude, and perception scores, were summarized by mean and standard deviation. For each questionnaire item, the frequency distributions of the responses were calculated (correct/incorrect for knowledge questions and 5-point Likert scale for attitude and perception). Differences between participant groups (e.g., sex, age group, and professional role) were assessed using the chi-square test or Fisher’s exact test for categorical variables and the Kruskal–Wallis test or univariate ANOVA for continuous variables, according to data distribution and the appropriateness of the tests. A *p*-value < 0.05 was considered statistically significant.

## 3. Results

### 3.1. Sociodemographic Data

Data collection was conducted between November 2024 and June 2025, with a total of 228 participants that completed the questionnaire. The sample analyzed was almost equally divided into males and females and divided between general dentists, pediatric dentistry residents and pediatric residents. Most participants were aged between 25 and 35 years. Moreover, results are presented by categorizing participants into three groups based on their professional background and in two groups based on their age and year of graduation, the first group including participants in the first 10 years of their postgraduate careers and another group including those in a later career stage, who graduated at least 10 years ago (Table 4).

### 3.2. Knowledge

The overall level of knowledge regarding oral manifestations associated with IBD varied considerably across respondents, as shown in Table 5. The vast majority (94.7%) correctly identified that IBDs have a higher prevalence in developed countries. Similarly, 93.4% knew that extraintestinal manifestations can precede gastrointestinal symptoms by several years. A high proportion (98.7%) was aware that IBD can also develop in children even before the second decade of life. However, substantial gaps emerged in other areas; in fact, only 11.4% knew that more than 10–20% of pediatric patients may present oral lesions. Regarding specific oral manifestations, 42.5% wrongly identified sialorrhea as a potential symptom, while 60.1% recognized angular cheilitis, and only 45.6% knew about parotid involvement with edema and ductal obstruction. Additionally, 50.4% wrongly responded that gingival hyperplasia is common in patients with ulcerative colitis, and 56.1% mistakenly considered oral keratosis an EIM of IBD. Comparative analyses showed statistically significant differences by professional role. Pediatric dentistry residents and pediatric residents achieved higher correct response rates on items concerning pediatric oral manifestations (*p* = 0.01). General dentists were less likely to correctly identify the prevalence of specific conditions such as pyostomatitis vegetans (64.9%) or the increased risk of oral cancer associated with immunosuppressive therapies (56.6%). No significant differences were observed between male and female respondents for most items, except for the association between immunosuppressive treatment and oral cancer risk, where females demonstrated higher awareness (*p* = 0.04). Participants aged ≤35 years tended to score higher on global knowledge than those over 45, although this difference did not reach statistical significance (*p* = 0.07).

### 3.3. Attitude

Participants’ self-perceived competence regarding the recognition and management of oral manifestations in IBD was heterogeneous, as detailed in Table 6. While 83% indicated they would refer patients to a specialist if suspect lesions were identified, only 39% felt confident in recognizing IBD-associated oral lesions, and even less participants (30%) expressed confidence in managing these manifestations in clinical practice. Additionally, 89% agreed on the importance of participating in continuing education programs, and 90% were inclined to update their knowledge by reading the scientific literature. Regarding the statement “I feel comfortable discussing IBD and oral manifestations with patients,” only 26.3% responded with “agree” or “strongly agree,” while 56.1% reported neutral or disagreeing responses, highlighting a lack of confidence in communication with the patient. Significant differences emerged among professional groups: pediatric residents and pediatric dentistry residents reported higher confidence in both recognizing and managing lesions compared to general dentists (*p* = 0.01). Furthermore, participants over 45 years of age were motivated to update their knowledge (*p* = 0.03). No significant differences were detected between sexes in perceived competence or inclination to collaborate with other professionals.

### 3.4. Perception

The distribution of responses (Table 7) shows that overall perception towards the importance of dentist involvement in IBD management was very positive. Regarding the statement “I think the dentist should inform patients about oral manifestations,” 82.1% responded with “agree” or “strongly agree,” with a mean score of 4.30 (SD = 0.801). A similar high level of agreement was observed for the statement “I believe dentists should collaborate with pediatricians and gastroenterologists,” with 96.5% agreeing or strongly agreeing. Moreover, 89.5% of participants agreed that dentists have an important role in the early diagnosis of IBD, but at the same time, 65% disagreed or strongly disagreed that dentists are adequately trained to manage oral complications associated with IBD. When comparing groups by profession, pediatric residents and pediatric dentistry residents reported higher agreement scores with statements on multidisciplinary collaboration and regular monitoring of oral health (*p* = 0.001). Conversely, general dentists were the group that more frequently expressed doubts about their educational background and recognized the need for additional training (*p* = 0.02). No significant associations were found between perception and sex or age group.

## 4. Discussion

The oral cavity is a frequent site of extraintestinal IBD, affecting up to 50% of patients with Crohn’s disease. Oral involvement is even more common in children and up to 50% of IBD pediatric patients develop oral lesions as a primary sign of disease [17,30]. Nonetheless, considering the minor incidence of IBD, ranging from 7 to 21 per 100,000 per year in the most affected geographical areas, the chance of a general dentist or a primary care pediatrician encountering a patient with oral manifestations of IBD is relatively low. On the other hand, the number of IBD prevalent cases is rapidly rising, especially among the pediatric population [11]. For this reason, the involvement of dentists in early detection and multidisciplinary management of IBD is becoming increasingly important. Our findings reveal relevant insights into current educational gaps and professional attitudes and perceptions regarding the role of the dentist in the care of patients with IBD.

Our study reported a clear disparity between general awareness of IBD and knowledge regarding its oral presentations. This aligns with the literature showing that awareness of oral manifestations of systemic diseases, including, but not limited to, IBD, is generally limited, even among trained healthcare professionals, despite the well-established link between oral and systemic health [33,34]. While most participants were, in fact, able to respond to general items, such as epidemiological questions, with an overall proportion of 74.4% correct answers, oral lesion specific knowledge was shown to be deficient, with just 58.2% correct answers. Primary care physicians and general dentists are not expected to possess exhaustive knowledge of all medical conditions and should not be overburdened with specialized diagnostic or therapeutic knowledge. Nonetheless, being aware of the oral manifestations of systemic conditions and of the referral pattern to specialized centers significantly increases the possibility of an early identification of the disease, consistently expediting patients’ diagnostic and therapeutic work-up. This is confirmed by a previous study that has underlined a correlation between the lack of knowledge and confidence of physicians and general dentists toward oral specific manifestations and a significant delay in patients’ referral and final diagnosis [35].

Additionally, just half of the participants were found to be aware of the association between immunosuppressive therapy and the risk of malignancies. Such a lack of knowledge may contribute to delayed diagnosis of oral cancer, markedly impacting patients’ quality of life and longevity as suggested by previous research [36,37].

The gap between pediatric dentistry residents and general dentists underlines the need for broader integration of pediatric and systemic pathology in dental curricula.

Furthermore, the trend toward higher scores among younger professionals, although not statistically significant, may indicate the gradual evolution of curricula toward more integrative approaches in recent years. However, this generational difference also highlights the need to offer structured continuing education for professionals already in practice, particularly those who may have received more traditional or siloed training.

Despite a general openness to interdisciplinary collaboration, participants showed uncertainty and lack of confidence regarding their clinical competence, particularly in both identifying and managing oral manifestations of IBD. Even lower was the proportion of participants who felt comfortable discussing these issues with patients. Communication challenges are particularly evident among younger professionals, who often experience heightened emotional involvement and uncertainty about their knowledge, both of which can undermine their confidence in interacting with patients, as reported in the literature [38]. This difference between theoretical awareness and clinical confidence may reflect a training model that emphasizes passive knowledge acquisition over applied practice and communication skills. Importantly, the higher self-confidence observed among pediatric dentistry residents and pediatric residents further supports the idea that clinical exposure is a key factor in building perceived competence.

The reduced willingness among older professionals to engage in continued learning points to a generational challenge: while younger practitioners may actively seek knowledge updates, targeted strategies may be required to engage more experienced clinicians, possibly by integrating learning into routine clinical settings. The large number of respondents interested in continuing education and keeping up with the scientific literature offers an important opportunity to improve clinical readiness. However, to fully realize this potential, educational strategies should prioritize case-based learning as a central tool, supported by actionable knowledge and interdisciplinary collaboration. This approach has proven effective in strengthening both the knowledge base and the critical thinking skills of dental trainees [39]. Moreover, evidence suggests that combining case-based and problem-based learning, despite their different focuses, can promote a more integrated and efficient learning process in dental curricula [40]. Recent studies have further emphasized that incorporating situational simulation [41] and role play within case-based frameworks [42] can significantly enhance non-operational clinical competencies, critical thinking, and teamwork, thereby reinforcing both diagnostic confidence and communication skills.

While clinical confidence was limited, the overall perception was generally positive, except for the statement regarding dentists’ educational background enabling them to manage oral complications in patients with IBD, which most participants disagreed with.

Positive attitudes toward interdisciplinary collaboration and a clear awareness of their professional role within the care team offer a constructive starting point for targeted educational interventions. Most agreed on the importance of collaboration between dentists, pediatricians and gastroenterologists and expressed the opinion that dentists should gain more knowledge on this topic during their dental training. This suggests that barriers to interdisciplinary work are more likely related to training and organization than to mindset. In fact, according to de Mendonça et al. [43], although interprofessional education is included in many dental programs, its integration is often limited in scope and not consistently supported by structured outcome assessments. However, some curricula have adopted more comprehensive approaches, embedding interprofessional education throughout multiple components of dental training to foster both professional and collaborative competencies in line with real-world healthcare needs [44].

General dentists were slightly less likely to agree that the oral health of patients with IBD should be regularly monitored to prevent complications and more often reported feeling unprepared. This highlights how a sense of unpreparedness may lead general dentists to undervalue the importance of monitoring oral health in IBD care. The fact that perception did not vary significantly by sex or age is also encouraging, as it shows that this openness is shared across demographics, a key advantage when planning broad educational initiatives.

In line with other survey-based studies, this work faces some limitations. Firstly, although the required sample size was achieved, the participants may not be fully representative of the broader target population. The use of self-reported data introduces potential response biases, particularly in items exploring attitudes and perceived responsibility, where participants might have provided answers they believed to be more acceptable or expected. Furthermore, the voluntary nature of participation may have led to a selection bias, favoring individuals with a pre-existing interest in IBD or oral–systemic health. While this may limit the study’s generalizability, it is noteworthy that relevant gaps in knowledge and confidence were observed even within this subgroup. Finally, another limitation of this study is the absence, in our questionnaire, of an item addressing the number of pediatric patients and IBD-related oral manifestations encountered during participants’ careers. These factors may potentially influence participants’ knowledge of the topic, as well as their responses regarding clinical attitudes and perceptions. Therefore, future studies addressing this limitation are needed to confirm our findings.

To conclude, this study highlights a promising readiness among pediatric residents, pediatric dentistry residents and general dentists to address the oral health aspects of IBD, especially in pediatric patients. While attitudes toward interdisciplinary care are highly favorable, substantial knowledge gaps remain, particularly regarding specific oral manifestations, and a lack of confidence is perceived in clinical management of IBD-related oral health and in communicating with patients with IBD. Despite the limited number of patients with IBD that a dentist or a primary care pediatrician could encounter in their own career, targeted education interventions focused on improving awareness, practical recognition of clinically relevant signs, communication, and appropriate referral pathways are suggested by our findings, especially in consideration of IBD’s increasing burden in the pediatric population.

## Figures and Tables

**Table 1:** Questionnaire assessing knowledge of oral lesions in patients with IBD. The statements required one of the following responses: “true”/“false”.

Knowledge on Oral Lesions in IBD
Chronic inflammatory bowel diseases (IBD) have a higher prevalence in developed countries compared to developing countries.
2. In the pediatric population affected by IBD, 10% to 20% of patients may present with oral manifestations.
3. IBD can develop in children only after the first decade of life.
4. The presence of extraintestinal manifestations in patients with IBD may indicate a higher level of inflammatory activity.
5. Extraintestinal manifestations of IBD can be the only clinical manifestation and sometimes precede gastrointestinal symptoms by years.
6. Oral extraintestinal manifestations of IBD in adults are more common than in the pediatric population.
7. Oral extraintestinal manifestations of IBD are classified as granulomatous diseases.
8. The most frequently affected site in oral extraintestinal lesions related to Crohn’s disease is the lips.
9. Oral lesions are more common in patients with Ulcerative Colitis compared to patients with Crohn’s disease.
10. As oral extraintestinal manifestations, patients with IBD may present with lip swelling.
11. As oral extraintestinal manifestations, patients with IBD may present with aphthous ulcers and deep linear ulcerations.
12. As oral extraintestinal manifestations, patients with IBD may present with sialorrhea.
13. As oral extraintestinal manifestations, patients with IBD may present with angular cheilitis.
14. As oral extraintestinal manifestations, patients with IBD may present with cobblestone appearance of the oral mucosa.
15. As oral extraintestinal manifestations, patients with IBD may present with oral keratosis.
16. As oral extraintestinal manifestations, patients with IBD may have parotid involvement with edema and ductal occlusion.
17. As oral extraintestinal manifestations, patients with IBD may present with gingivitis and periodontal disease.
18. Gingivitis associated with IBD is more refractory to therapy commonly used for plaque-related classic gingivitis, such as proper oral hygiene or professional oral hygiene sessions.
19. In patients with IBD, the prevalence of periodontal disease is estimated to be higher than 30%.
20. Among the oral extraintestinal manifestations is vegetative pyostomatitis.
21. As oral extraintestinal manifestations, ulcers always disappear during periods of intestinal remission.
22. As an oral extraintestinal manifestation, gingival hyperplasia is very common in patients with ulcerative colitis (UC).
23. The prevalence of oral extraintestinal manifestations is higher in patients in remission compared to those in the active phase.
24. Individuals with IBD on immunosuppressive therapy have a higher risk of developing oral cancer.
25. Some oral extraintestinal manifestations may be a consequence of pharmacological treatment for IBD.
26. The extraintestinal manifestations of IBD have no correlation with malnutrition.
27. Usually, the oral extraintestinal manifestations of IBD respond well to the medical therapy used for the treatment of the systemic disease.
28. The prevalence of oral manifestations is higher in patients in remission compared to those in the active phase.
29. Among oral extraintestinal manifestations, vegetative pyostomatitis is more common in patients with Crohn’s disease than in those with ulcerative colitis.
30. There are no topical treatments for oral extraintestinal manifestations, and only treatment for the systemic disease can be performed.

**Table 2:** Questionnaire assessing participants’ attitudes toward oral lesions in patients with IBD. Agreement with each statement was measured using a 5-point Likert scale: 1, strongly disagree; 2, disagree; 3, neither agree nor disagree; 4, agree; 5, strongly agree.

Attitude
Based on my knowledge, I feel comfortable discussing IBD and its oral manifestations with patients/parents.
2. I believe I can recognize an oral lesion related to IBD when I see one.
3. I believe I know how to manage oral lesions in patients with IBD and can apply this knowledge in practice.
4. I am likely to advise my patients to consult a gastroenterologist or pediatrician if I detect suspicious lesions.
5. I think dentists should attend conferences/seminars related to IBD.
6. I am inclined to personally update my knowledge about IBD by reading journals and scientific articles.
7. I believe that oral lesions in patients with IBD should be treated using a multidisciplinary approach.
8. I am willing to invest time in learning more about IBD during my dental career.
9. I am inclined to collaborate with gastroenterologists and pediatricians to provide more comprehensive care for patients with IBD.
10. I am inclined to promote awareness of oral lesions related to IBD among my colleagues.

**Table 3:** Questionnaire assessing participants’ perceptions of oral lesions in patients with IBD. Agreement with each statement was measured using a 5-point Likert scale: 1, strongly disagree; 2, disagree; 3, neither agree nor disagree; 4, agree; 5, strongly agree.

Perception
I believe the dentist has an important role in the early diagnosis of IBD.
2. I think the dentist should inform patients about oral manifestations associated with IBD.
3. I believe the dentist plays a significant role in managing oral extraintestinal manifestations in patients with IBD.
4. I think the dentist should collaborate with gastroenterologists and pediatricians in managing patients with IBD.
5. I need more information about the oral complications of IBD.
6. I need further information on the treatment of oral manifestations of IBD.
7. I believe parents of patients are interested in receiving information about the oral manifestations of IBD.
8. I think dental students should acquire more knowledge about IBD and its oral extraintestinal manifestations during their dental education.
9. I believe dentists are adequately prepared to manage oral complications associated with IBD.
10. I think the oral health of patients with IBD should be regularly monitored by the dentist to prevent complications.

**Table 4:** Sociodemographic analysis of respondents (*N* = 228) and percentage of correct responses according to sex, age range, graduation year and geographical region.

Categories	Number of Participants	% Correct Answers
Sex		
Female	107/228 (46.9%)	58.2%
Male	121/228 (53.1%)	61.4%
Age group		
25–35 years	135/228 (59.2%)	63%
>35 years	93/228 (40.8%)	58.7%
Profession		
General dentists	164/228 (72%)	63%
Pediatric dental residents	34/228 (15%)	64.4%
Pediatric residents	30/228 (13%)	70.4%
Years since graduation		
<10 years	134/228 (58.8%)	63.4%
≥10 years	94/228 (41.2%)	56.8%
Geographical region		
North/Central	63/228 (27.6%)	62.7%
South/Islands	165/228 (72.4%)	58.6%

**Table 5:** Responses to the knowledge section of the questionnaire reported as a percentage of correct and incorrect answers per item.

Item	Correct (%)	Incorrect (%)
IBDs have higher prevalence in developed countries	94.7	5.3
More than 10–20% of pediatric patients may have oral manifestations	11.4	88.6
IBD can arise before the second decade of life	98.7	1.3
Extraintestinal manifestations can precede gastrointestinal symptoms	93.4	6.6
Extraintestinal oral manifestations are more frequent in adults	66.2	33.8
Oral manifestations are granulomatous diseases	46.9	53.1
Lips are most frequently involved in CD	44.3	55.7
Oral lesions more frequent in UC	69.3	30.7
Tumefaction of the lips is a manifestation	73.7	26.3
Ulcerations and aphthae are manifestations	83.3	16.7
Sialorrhea is a manifestation	57.5	42.5
Angular cheilitis is amanifestation	60.1	39.9
Cobblestoning of oral mucosa	67.1	32.9
Oral keratosis	43.9	56.1
Parotid involvement	45.6	54.4
Gingivitis and periodontitis are manifestations	82.5	17.5
Gingivitis is more refractory to therapy	81.1	18.9
Periodontal disease prevalence is >30%	46.5	53.5
Pyostomatitis vegetans is a manifestation	64.9	35.1
Ulcers always disappear during remission	66.2	33.8
Gingival hyperplasia are more common in ulcerative colitis	49.6	50.4
Oral manifestations are more frequent in remission	75.0	25.0
Immunosuppressed patients have higher oral cancer risk	56.6	43.4
Oral lesions may result from drug therapy	71.1	28.9
Oral manifestations are unrelated to malnutrition	90.8	9.2
Oral lesions respond well to systemic therapy	32.9	67.1
Oral manifestations are more frequent in remission	81.1	18.9
Pyostomatitis vegetans is more frequent in CD	50.4	49.6
No topical treatment is available	79.8	20.2

**Table 6:** Responses to the attitude section of the questionnaire concerning recognition, management and communication of oral lesions, based on a 5-point Likert scale.

Item	1 (%)	2 (%)	3 (%)	4 (%)	5 (%)
Confident in recognizing lesions	4.4	36.8	17.1	39.0	2.6
Confident in managing lesions	11.4	44.3	24.1	18.4	1.8
Comfortable discussing with patients	19.3	36.8	17.5	23.7	2.6
Inclined to refer to specialists	–	1.8	9.2	49.6	39.5
Willing to update knowledge by reading	–	–	11.8	49.1	39.0
Willing to invest time in learning	0.4	–	17.5	49.1	32.9
Inclined to collaborate with other professionals	–	–	1.8	34.2	64.0
Willing to promote awareness	–	–	4.8	41.2	53.9

**Table 7:** Responses to the perception section of the questionnaire concerning recognition, management and communication of oral lesions, based on a 5-point Likert scale.

Item	1 (%)	2 (%)	3 (%)	4 (%)	5 (%)
Dentists have an important role in early diagnosis	–	–	11.0	45.2	43.9
Dentists should inform patients about manifestations	–	1.8	16.2	32.5	49.6
Dentists should collaborate with pediatricians/gastroenterologists	–	–	3.5	28.5	68.0
Dentists have a relevant role in management	0.4	–	7.0	42.1	50.4
Oral health should be regularly monitored	–	2.2	5.7	64.9	27.2
Dental students need more IBD training during university training	–	–	14.9	33.3	51.8
Dentists are adequately trained to manage oral IBD-related manifestations	18.9	46.1	12.7	19.7	2.6

## Data Availability

The original contributions presented in this study are included in the article/Supplementary Materials. Further inquiries can be directed to the corresponding author.

## Supplementary Materials

The following supporting information can be downloaded at: https://www.mdpi.com/article/10.3390/jcm15041598/s1, Text S1: Questionnaire ita version; Text S2: Questionnaire eng version.

## Abbreviations

The following abbreviations are used in this manuscript:

KAP	Knowledge, Attitude and Perception
IBD	Inflammatory Bowel Disease
CD	Crohn’s Disease
UC	Ulcerative Colitis
EIMs	Extraintestinal Manifestations
