# Supplementary material for: The Knowledge, Attitude, and Perception (KAP) of Healthcare Professionals in Pediatric Settings Toward Oral Manifestations of Inflammatory Bowel Disease (IBD): A Survey-Based Cross-Sectional Study
Source: J Clin Med. 2026 Feb 19;15(4):1598. doi: 10.3390/jcm15041598 (PMC12941399; doi:10.3390/jcm15041598)
Supplement: Supplementary file 1 [file jcm-15-01598-s001.zip › Text S1.pdf]

Variabili socio-demografiche

1. Sesso: M/F
2. Età:
3. Professione: odontoiatra generico/odontoiatra pediatrico/ specializzando in pedodonzia/specializzando in pediatria/altro
4. Anno di conseguimento della laurea:
5. Università di conseguimento della laurea:
6. Regione geografica in cui si pratica l’attività:
7. Hai mai visto un paziente con MICI?:
a. Se si, quanti?
8. Hai mai trattato un paziente con MICI?:
a. Se si, quanti?

Knowledge (Risposte dicotomiche Vero/Falso)

1. Le Malattie infiammatorie croniche intestinali (MICI) hanno una prevalenza più alta nei paesi sviluppati rispetto a quelli in via di sviluppo.
2. Nella popolazione pediatrica affetta da MICI, dal 10% al 20% dei pazienti può presentare manifestazioni orali.
3. Le MICI possono insorgere nei bambini solo dopo la prima decade di vita.
4. La presenza di manifestazioni extraintestinali nei pazienti affetti da MICI può essere indice di una maggiore attività infiammatoria.
5. Le manifestazioni extraintestinali di MICI possono essere l'unica manifestazione clinica e talvolta precedere i sintomi gastrointestinali anche di anni.
6. Le manifestazioni extraintestinali orali delle MICI negli adulti sono più frequenti rispetto alla popolazione pediatrica.
7. Le manifestazioni extraintestinali orali di MICI rientrano nella categoria delle malattie granulamotosiche.
8. Il sito più frequentemente colpito dalle lesioni extraintestinali orali correlate alla malattia di Chron sono le labbra.
9. Le lesioni orali sono più frequenti nei pazienti con Colite Ulcerosa rispetto ai pazienti con Malattia di Chron.
10. Come manifestazioni extraintestinali orali, i pazienti affetti da MICI possono presentare tumefazione labiale.
11. Come manifestazioni extraintestinali orali, i pazienti affetti da MICI possono presentare ulcere aftosiche e ulcerazioni lineari profonde.
12. Come manifestazioni extraintestinali orali, i pazienti affetti da MICI possono presentare scialorrea.
13. Come manifestazioni extraintestinali orali, i pazienti affetti da MICI possono presentare cheilite angolare.
14. Come manifestazioni extraintestinali orali, i pazienti affetti da MICI possono presentare l’aspetto cobblestoning della mucosa orale.
15. Come manifestazioni extraintestinali orali, i pazienti affetti da MICI possono presentare cheratosi orali.
16. Come manifestazioni extraintestinali orali, nei pazienti affetti da MICI ci può essere un interessamento della parotide con edema ed occlusione duttale.
17. Come manifestazioni extraintestinali orali, i pazienti affetti da MICI possono presentare gengivite e malattia parodontale.
18. La gengivite associata a MICI risulta essere maggiormente refrattaria alla terapia comunemente utilizzate nella gengivite classica placca-correlata, come la corretta igiene orale, o sedute di igiene orale professionale.
19. Nei pazienti affetti da MICI la prevalenza della malattia parodontale è stimata essere superiore al 30%.
20. Tra le manifestazioni extraintestinali orali rientra la piostomatite vegetante.
21. Come manifestazioni extraintestinali orali, le ulcere scompaiono sempre nei periodi di remissione intestinale.

22. Come manifestazione extraintesinale orale, l'iperplasia gengivale è una manifestazione molto comune nei pazienti con colite ulcerosa.
23. La prevalenza delle manifestazioni extraintestinali orali è maggiore nei pazienti in remissione rispetto a quelli in fase attiva.
24. Le persone affette da MICI, in terapia immunosoppressiva, hanno un maggiore rischio di sviluppare cancro orale.
25. Alcune manifestazioni extraintestinali orali possono essere una conseguenza del trattamento farmacologico delle MICI.
26. Le manifestazioni extraintesinali delle MICI non hanno alcuna correlazione con la malnutrizione.
27. Di solito le manifestazioni extraintestinali orali delle MICI rispondono bene alla terapia medica adoperata per il trattamento della patologia sistemica.
28. La prevalenza delle manifestazioni orali è maggiore nei pazienti in remissione rispetto a quelli in fase attiva.
29. Tra le manifestazioni orali extraintestinali, la piostomatite vegetante è più frequente nei pazienti affetti da morbo di Chron rispetto a quelli affetti da colite ulcerosa.
30. Per il trattamento delle manifestazioni extraintestinali orali non esistono trattamenti topico, ma può essere eseguito solo il trattamento della patologia sistemica.

Attitude (Scala Likert a 5 punti)

1. In base alle mie conoscenze, mi sento a mio agio nel discutere di MICI e delle sue manifestazioni orali con pazienti/genitori.
   1. Completamente in disaccordo 2. In disaccordo 3. Né in disaccordo né d'accordo 4. D'accordo 5. Completamente d'accordo
2. Ritengo di essere in grado di riconoscere una lesione orale correlata alla MICI quando la vedo.
   1. Completamente in disaccordo 2. In disaccordo 3. Né in disaccordo né d'accordo 4. D'accordo 5. Completamente d'accordo
3. Ritengo di sapere come gestire le lesioni orali nei pazienti con MICI e di poter applicare queste conoscenze nella pratica clinica.
   1. Completamente in disaccordo 2. In disaccordo 3. Né in disaccordo né d'accordo 4. D'accordo 5. Completamente d'accordo
4. È probabile che consigli ai miei pazienti di consultare un gastroenterologo o un pediatra se rilevo lesioni sospette.
   1. Completamente in disaccordo 2. In disaccordo 3. Né in disaccordo né d'accordo 4. D'accordo 5. Completamente d'accordo
5. Penso che i dentisti dovrebbero partecipare a conferenze/seminari relativi alle MICI.
   1. Completamente in disaccordo 2. In disaccordo 3. Né in disaccordo né d'accordo 4. D'accordo 5. Completamente d'accordo
6. Sono incline ad aggiornare personalmente le mie conoscenze sulle MICI leggendo riviste e articoli scientifici.
   1. Completamente in disaccordo 2. In disaccordo 3. Né in disaccordo né d'accordo 4. D'accordo 5. Completamente d'accordo
7. Ritengo che le lesioni orali nei pazienti con MICI debbano essere trattate con un approccio multidisciplinare.
   1. Completamente in disaccordo 2. In disaccordo 3. Né in disaccordo né d'accordo 4. D'accordo 5. Completamente d'accordo

8. Sono disposto/a a investire tempo per approfondire le mie conoscenze sulle MICI durante la mia carriera odontoiatrica.
   1. Completamente in disaccordo 2. In disaccordo 3. Né in disaccordo né d'accordo 4. D'accordo 5. Completamente d'accordo
9. Sono incline a collaborare con gastroenterologi e pediatri per offrire un'assistenza più completa ai pazienti con MICI.
   1. Completamente in disaccordo 2. In disaccordo 3. Né in disaccordo né d'accordo 4. D'accordo 5. Completamente d'accordo
10. Sono incline a promuovere la consapevolezza delle lesioni orali correlate alle MICI tra i miei colleghi.
   1. Completamente in disaccordo 2. In disaccordo 3. Né in disaccordo né d'accordo 4. D'accordo 5. Completamente d'accordo

Perception (Scala Likert a 5 punti)

1. Ritengo che il dentista abbia un ruolo importante nella diagnosi precoce delle MICI.
   1. Completamente in disaccordo 2. In disaccordo 3. Né in disaccordo né d'accordo 4. D'accordo 5. Completamente d'accordo
2. Penso che il dentista debba informare i pazienti sulle manifestazioni orali associate alle MICI.
   1. Completamente in disaccordo 2. In disaccordo 3. Né in disaccordo né d'accordo 4. D'accordo 5. Completamente d'accordo
3. Ritengo che il dentista svolga un ruolo significativo nella gestione delle manifestazioni extraintestinali orali nei pazienti con MICI.
   1. Completamente in disaccordo 2. In disaccordo 3. Né in disaccordo né d'accordo 4. D'accordo 5. Completamente d'accordo
4. Penso che il dentista debba collaborare con gastroenterologi e pediatri nella gestione dei pazienti con MICI.
   1. Completamente in disaccordo 2. In disaccordo 3. Né in disaccordo né d'accordo 4. D'accordo 5. Completamente d'accordo
5. Ho bisogno di maggiori informazioni sulle complicanze orali delle MICI.
   1. Completamente in disaccordo 2. In disaccordo 3. Né in disaccordo né d'accordo 4. D'accordo 5. Completamente d'accordo
6. Ho bisogno di ulteriori informazioni sul trattamento delle manifestazioni orali delle MICI.
   1. Completamente in disaccordo 2. In disaccordo 3. Né in disaccordo né d'accordo 4. D'accordo 5. Completamente d'accordo
7. Ritengo che i genitori dei pazienti siano interessati a ricevere informazioni sulle manifestazioni orali delle MICI.
   1. Completamente in disaccordo 2. In disaccordo 3. Né in disaccordo né d'accordo 4. D'accordo 5. Completamente d'accordo
8. Penso che gli studenti di odontoiatria dovrebbero acquisire maggiori conoscenze sulle MICI e sulle loro manifestazioni orali extraintestinali durante il percorso formativo.
   1. Completamente in disaccordo 2. In disaccordo 3. Né in disaccordo né d'accordo 4. D'accordo 5. Completamente d'accordo
9. Ritengo che i dentisti siano adeguatamente preparati a gestire le complicanze orali associate alle MICI.
   1. Completamente in disaccordo 2. In disaccordo 3. Né in disaccordo né d'accordo 4. D'accordo 5. Completamente d'accordo
10. Penso che la salute orale dei pazienti con MICI debba essere regolarmente monitorata dal dentista per prevenire complicanze.

1. Completamente in disaccordo 2. In disaccordo 3. Né in disaccordo né d’accordo 4. D’accordo 5. Completamente d’accordo

References

1. Lankarani KB, Sivandzadeh GR, Hassanpour S. Oral manifestation in inflammatory boweldisease: a review. World J Gastroenterol. 2013 Dec 14;19(46):8571-9. doi: 10.3748/wjg.v19.i46.8571. PMID: 24379574; PMCID: PMC3870502.
2. Agrawal M, Jess T. Implications of the changing epidemiology of inflammatory bowel disease in a changing world. United European Gastroenterol J. 2022 Dec;10(10):1113-1120. doi: 10.1002/ueg2.12317. Epub 2022 Oct 17. PMID: 36251359; PMCID: PMC9752308.
3. Kaplan GG. The global burden of IBD: from 2015 to 2025. Nat Rev Gastroenterol Hepatol. 2015 Dec;12(12):720-7. doi: 10.1038/nrgastro.2015.150. Epub 2015 Sep 1. PMID: 26323879.
4. Shazib MA, Byrd KM, Gulati AS. Diagnosis and Management of Oral Extraintestinal Manifestations of Pediatric Inflammatory Bowel Disease. J Pediatr Gastroenterol Nutr. 2022 Jan 1;74(1):7-12. doi: 10.1097/MPG.0000000000003302. PMID: 34560727; PMCID: PMC8714692.
5. Papageorgiou SN, Hagner M, Nogueira AV, Franke A, Jäger A, Deschner J. Inflammatory bowel disease and oral health: systematic review and a meta-analysis. J Clin Periodontol. 2017 Apr;44(4):382-393. doi: 10.1111/jcpe.12698. Epub 2017 Mar 6. PMID: 28117909.
6. Lauritano D, Boccalari E, Di Stasio D, Della Vella F, Carinci F, Lucchese A, Petruzzi M. Prevalence of Oral Lesions and Correlation with Intestinal Symptoms of Inflammatory BowelDisease: A Systematic Review. Diagnostics (Basel). 2019 Jul 15;9(3):77. doi: 10.3390/diagnostics9030077. PMID: 31311171; PMCID: PMC6787704.
7. Vernon-Roberts A, Day AS. Promoting early testing and appropriate referral to reduce diagnostic delay for children with suspected inflammatory bowel disease, a narrative review. TranslPediatr. 2023 Jul 31;12(7):1416-1430. doi: 10.21037/tp-23-35. Epub 2023 Jun 25. PMID: 37575896; PMCID: PMC10416131
8. Rosen MJ, Dhawan A, Saeed SA. Inflammatory Bowel Disease in Children and Adolescents. JAMA Pediatr. 2015 Nov;169(11):1053-60. doi: 10.1001/jamapediatrics.2015.1982. PMID: 26414706; PMCID: PMC4702263.
9. Jang HJ, Kang B, Choe BH. The difference in extraintestinal manifestations of inflammatory bowel disease for children and adults. Transl Pediatr. 2019 Jan;8(1):4-15. doi: 10.21037/tp.2019.01.06. PMID: 30881893; PMCID: PMC6382501
10. Mawardi H, Alsubhi A, Salem N, Alhadlaq E, Dakhil S, Zahran M, Elbadawi L. Management of medication-induced gingival hyperplasia: a systematic review. Oral Surg Oral Med Oral Pathol Oral Radiol. 2021 Jan;131(1):62-72. doi: 10.1016/j.oooo.2020.10.020. Epub 2020 Oct26. PMID: 33214091.
11. Katsanos KH, Torres J, Roda G, Brygo A, Delaporte E, Colombel JF. Review article: non-malignant oral manifestations in inflammatory bowel diseases. Aliment Pharmacol Ther. 2015 Jul;42(1):40-60. doi: 10.1111/apt.13217. Epub 2015 Apr 28. PMID: 25917394
12. Malins TJ, Wilson A, Ward-Booth RP. Recurrent buccal space abscesses: a complication of Crohn's disease. Oral Surg Oral Med Oral Pathol. 1991 Jul;72(1):19-21. doi: 10.1016/0030-4220(91)90182-c. PMID: 1891238.
13. Femiano F, Lanza A, Buonaiuto C, Perillo L, Dell'Ermo A, Cirillo N. Pyostomatitis vegetans: a review of the literature. Med Oral Patol Oral Cir Bucal. 2009 Mar 1;14(3):E114-7. PMID: 19242389.
14. Pittock S, Drumm B, Fleming P, McDermott M, Imrie C, Flint S, Bourke B. The oral cavity in Crohn's disease. J Pediatr. 2001 May;138(5):767-71. doi: 10.1067/mpd.2001.113008. PMID: 11343060.
15. Harty S, Fleming P, Rowland M, Crushell E, McDermott M, Drumm B, Bourke B. A prospective study of the oral manifestations of Crohn's disease. Clin Gastroenterol Hepatol. 2005 Sep;3(9):886-91. doi: 10.1016/s1542-3565(05)00424-6. PMID: 16234026.
16. Jang HJ, Kang B, Choe BH. The difference in extraintestinal manifestations of inflammatory bowel disease for children and adults. Transl Pediatr. 2019 Jan;8(1):4-15. doi: 10.21037/tp.2019.01.06. PMID: 30881893; PMCID: PMC6382501.
17. Long D, Wang C, Huang Y, Mao C, Xu Y, Zhu Y. Changing epidemiology of inflammatory bowel disease in children and adolescents. Int J Colorectal Dis. 2024 May 18;39(1):73. doi: 10.1007/s00384-024-04640-9. PMID: 38760622; PMCID: PMC11101569.
18. Katsanos KH, Roda G, Brygo A, Delaporte E, Colombel JF. Oral Cancer and Oral Precancerous Lesions in Inflammatory Bowel Diseases: A Systematic Review. J Crohns Colitis. 2015 Nov;9(11):1043-52. doi: 10.1093/ecco-jcc/jjv122. Epub 2015 Jul 10. PMID: 26163301.

19. Lourenço SV, Hussein TP, Bologna SB, Sipahi AM, Nico MM. Oral manifestations of inflammatory bowel disease: a review based on the observation of six cases. J Eur AcadDermatol Venereol. 2010 Feb;24(2):204-7. doi: 10.1111/j.1468-3083.2009.03304.x. Epub 2009 Jun 22. PMID: 19552719.
20. Conrad MA, Bittinger K, Ren Y, Kachelries K, Vales J, Li H, Wu GD, Bushman FD, Devoto M, Baldassano RN, Kelsen JR. The intestinal microbiome of inflammatory bowel disease across the pediatric age range. Gut Microbes. 2024 Jan-Dec;16(1):2317932. doi: 10.1080/19490976.2024.2317932. Epub 2024 Feb 25. PMID: 38404111; PMCID: PMC10900269.
21. Zbar AP, Ben-Horin S, Beer-Gabel M, Eliakim R. Oral Crohn's disease: is it a separable disease from orofacial granulomatosis? A review. J Crohns Colitis. 2012 Mar;6(2):135-42. doi: 10.1016/j.crohns.2011.07.001. Epub 2011 Aug 9. PMID: 22325167.
22. Grave B, McCullough M, Wiesenfeld D. Orofacial granulomatosis--a 20-year review. Oral Dis. 2009 Jan;15(1):46-51. doi: 10.1111/j.1601-0825.2008.01500.x. PMID: 19076470.
23. Veloso FT. Extraintestinal manifestations of inflammatory bowel disease: do they influence treatment and outcome? World J Gastroenterol. 2011 Jun 14;17(22):2702-7. doi: 10.3748/wjg.v17.i22.2702. PMID: 21734777; PMCID: PMC3122258.
