# Supplementary material for: The Knowledge, Attitude, and Perception (KAP) of Healthcare Professionals in Pediatric Settings Toward Oral Manifestations of Inflammatory Bowel Disease (IBD): A Survey-Based Cross-Sectional Study
Source: J Clin Med. 2026 Feb 19;15(4):1598. doi: 10.3390/jcm15041598 (PMC12941399; doi:10.3390/jcm15041598)
Supplement: Supplementary file 1 [file jcm-15-01598-s001.zip › Text S2.pdf]

Socio-demographic variables

1. Sex**:** M/F
2. Age**:**
3. Profession**:** General dentist / Pediatric dentist / Resident in pediatric dentistry / Resident in pediatrics / Other
4. Year of graduation:
5. University of graduation:
6. Geographical region where you practice:
7. Have you ever seen a patient with IBD?
    a. If yes, how many?
8. Have you ever treated a patient with IBD?
    a. If yes, how many?

Knowledge (dichotomous true/false responses)

1. Chronic inflammatory bowel diseases (IBD) have a higher prevalence in developed countries compared to developing countries.
2. In the pediatric population affected by IBD, 10% to 20% of patients may present with oral manifestations.
3. IBD can develop in children only after the first decade of life.
4. The presence of extraintestinal manifestations in patients with IBD may indicate a higher level of inflammatory activity.
5. Extraintestinal manifestations of IBD can be the only clinical manifestation and sometimes precede gastrointestinal symptoms by years.
6. Oral extraintestinal manifestations of IBD in adults are more common than in the pediatric population.
7. Oral extraintestinal manifestations of IBD are classified as granulomatous diseases.
8. The most frequently affected site in oral extraintestinal lesions related to Crohn's disease is the lips.
9. Oral lesions are more common in patients with Ulcerative Colitis compared to patients with Crohn's disease.
10. As oral extraintestinal manifestations, patients with IBD may present with lip swelling.
11. As oral extraintestinal manifestations, patients with IBD may present with aphthous ulcers and deep linear ulcerations.
12. As oral extraintestinal manifestations, patients with IBD may present with sialorrhea.
13. As oral extraintestinal manifestations, patients with IBD may present with angular cheilitis.
14. As oral extraintestinal manifestations, patients with IBD may present with cobblestone appearance of the oral mucosa.
15. As oral extraintestinal manifestations, patients with IBD may present with oral keratosis.
16. As oral extraintestinal manifestations, patients with IBD may have parotid involvement with edema and ductal occlusion.
17. As oral extraintestinal manifestations, patients with IBD may present with gingivitis and periodontal disease.

18. Gingivitis associated with IBD is more refractory to therapy commonly used for plaque-related classic gingivitis, such as proper oral hygiene or professional oral hygiene sessions.
19. In patients with IBD, the prevalence of periodontal disease is estimated to be higher than 30%.
20. Among the oral extraintestinal manifestations is vegetative pyostomatitis.
21. As oral extraintestinal manifestations, ulcers always disappear during periods of intestinal remission.
22. As an oral extraintestinal manifestation, gingival hyperplasia is very common in patients with ulcerative colitis.
23. The prevalence of oral extraintestinal manifestations is higher in patients in remission compared to those in the active phase.
24. Individuals with IBD on immunosuppressive therapy have a higher risk of developing oral cancer.
25. Some oral extraintestinal manifestations may be a consequence of pharmacological treatment for IBD.
26. The extraintestinal manifestations of IBD have no correlation with malnutrition.
27. Usually, the oral extraintestinal manifestations of IBD respond well to the medical therapy used for the treatment of the systemic disease.
28. The prevalence of oral manifestations is higher in patients in remission compared to those in the active phase.
29. Among oral extraintestinal manifestations, vegetative pyostomatitis is more common in patients with Crohn's disease than in those with ulcerative colitis.
30. There are no topical treatments for oral extraintestinal manifestations, and only treatment for the systemic disease can be performed.

Attitude (Likert scale - 5 points)

1. Based on my knowledge, I feel comfortable discussing IBD and its oral manifestations with patients/parents.
   1. Strongly disagree 2. Disagree 3. Neither agree nor disagree 4. Agree 5. Strongly agree
2. I believe I can recognize an oral lesion related to IBD when I see one.
   1. Strongly disagree 2. Disagree 3. Neither agree nor disagree 4. Agree 5. Strongly agree
3. I believe I know how to manage oral lesions in patients with IBD and can apply this knowledge in practice.
   1. Strongly disagree 2. Disagree 3. Neither agree nor disagree 4. Agree 5. Strongly agree
4. I am likely to advise my patients to consult a gastroenterologist or pediatrician if I detect suspicious lesions.
   1. Strongly disagree 2. Disagree 3. Neither agree nor disagree 4. Agree 5. Strongly agree
5. I think dentists should attend conferences/seminars related to IBD.
   1. Strongly disagree 2. Disagree 3. Neither agree nor disagree 4. Agree 5. Strongly agree
6. I am inclined to personally update my knowledge about IBD by reading journals and scientific articles.
   1. Strongly disagree 2. Disagree 3. Neither agree nor disagree 4. Agree 5. Strongly agree

7. I believe that oral lesions in patients with IBD should be treated using a multidisciplinary approach.
   1. Strongly disagree 2. Disagree 3. Neither agree nor disagree 4. Agree 5. Strongly agree
8. I am willing to invest time in learning more about IBD during my dental career.
   1. Strongly disagree 2. Disagree 3. Neither agree nor disagree 4. Agree 5. Strongly agree
9. I am inclined to collaborate with gastroenterologists and pediatricians to provide more comprehensive care for patients with IBD.
   1. Strongly disagree 2. Disagree 3. Neither agree nor disagree 4. Agree 5. Strongly agree
10. I am inclined to promote awareness of oral lesions related to IBD among my colleagues.
   1. Strongly disagree 2. Disagree 3. Neither agree nor disagree 4. Agree 5. Strongly agree

Perception (Likert scale - 5 points)

1. I believe the dentist has an important role in the early diagnosis of IBD.
   1. Strongly disagree 2. Disagree 3. Neither agree nor disagree 4. Agree 5. Strongly agree
2. I think the dentist should inform patients about oral manifestations associated with IBD.
   1. Strongly disagree 2. Disagree 3. Neither agree nor disagree 4. Agree 5. Strongly agree
3. I believe the dentist plays a significant role in managing oral extraintestinal manifestations in patients with IBD.
   1. Strongly disagree 2. Disagree 3. Neither agree nor disagree 4. Agree 5. Strongly agree
4. I think the dentist should collaborate with gastroenterologists and pediatricians in managing patients with IBD.
   1. Strongly disagree 2. Disagree 3. Neither agree nor disagree 4. Agree 5. Strongly agree
5. I need more information about the oral complications of IBD.
   1. Strongly disagree 2. Disagree 3. Neither agree nor disagree 4. Agree 5. Strongly agree
6. I need further information on the treatment of oral manifestations of IBD.
   1. Strongly disagree 2. Disagree 3. Neither agree nor disagree 4. Agree 5. Strongly agree
7. I believe parents of patients are interested in receiving information about the oral manifestations of IBD.
   1. Strongly disagree 2. Disagree 3. Neither agree nor disagree 4. Agree 5. Strongly agree
8. I think dental students should acquire more knowledge about IBD and its oral extraintestinal manifestations during their dental education.
   1. Strongly disagree 2. Disagree 3. Neither agree nor disagree 4. Agree 5. Strongly agree
9. I believe dentists are adequately prepared to manage oral complications associated with IBD.
   1. Strongly disagree 2. Disagree 3. Neither agree nor disagree 4. Agree 5. Strongly agree

10. I think the oral health of patients with IBD should be regularly monitored by the dentist to prevent complications.
    1. Strongly disagree 2. Disagree 3. Neither agree nor disagree 4. Agree 5. Strongly agree

References

1. Lankarani KB, Sivandzadeh GR, Hassanpour S. Oral manifestation in inflammatory boweldisease: a review. World J Gastroenterol. 2013 Dec 14;19(46):8571-9. doi: 10.3748/wjg.v19.i46.8571. PMID: 24379574; PMCID: PMC3870502.
2. Agrawal M, Jess T. Implications of the changing epidemiology of inflammatory bowel disease in a changing world. United European Gastroenterol J. 2022 Dec;10(10):1113-1120. doi: 10.1002/ueg2.12317. Epub 2022 Oct 17. PMID: 36251359; PMCID: PMC9752308.
3. Kaplan GG. The global burden of IBD: from 2015 to 2025. Nat Rev Gastroenterol Hepatol. 2015 Dec;12(12):720-7. doi: 10.1038/nrgastro.2015.150. Epub 2015 Sep 1. PMID: 26323879.
4. Shazib MA, Byrd KM, Gulati AS. Diagnosis and Management of Oral Extraintestinal Manifestations of Pediatric Inflammatory Bowel Disease. J Pediatr Gastroenterol Nutr. 2022 Jan 1;74(1):7-12. doi: 10.1097/MPG.0000000000003302. PMID: 34560727; PMCID: PMC8714692.
5. Papageorgiou SN, Hagner M, Nogueira AV, Franke A, Jäger A, Deschner J. Inflammatory bowel disease and oral health: systematic review and a meta-analysis. J Clin Periodontol. 2017 Apr;44(4):382-393. doi: 10.1111/jcpe.12698. Epub 2017 Mar 6. PMID: 28117909.
6. Lauritano D, Boccalari E, Di Stasio D, Della Vella F, Carinci F, Lucchese A, Petruzzi M. Prevalence of Oral Lesions and Correlation with Intestinal Symptoms of Inflammatory BowelDisease: A Systematic Review. Diagnostics (Basel). 2019 Jul 15;9(3):77. doi: 10.3390/diagnostics9030077. PMID: 31311171; PMCID: PMC6787704.
7. Vernon-Roberts A, Day AS. Promoting early testing and appropriate referral to reduce diagnostic delay for children with suspected inflammatory bowel disease, a narrative review. TranslPediatr. 2023 Jul 31;12(7):1416-1430. doi: 10.21037/tp-23-35. Epub 2023 Jun 25. PMID: 37575896; PMCID: PMC10416131
8. Rosen MJ, Dhawan A, Saeed SA. Inflammatory Bowel Disease in Children and Adolescents. JAMA Pediatr. 2015 Nov;169(11):1053-60. doi: 10.1001/jamapediatrics.2015.1982. PMID: 26414706; PMCID: PMC4702263.
9. Jang HJ, Kang B, Choe BH. The difference in extraintestinal manifestations of inflammatory bowel disease for children and adults. Transl Pediatr. 2019 Jan;8(1):4-15. doi: 10.21037/tp.2019.01.06. PMID: 30881893; PMCID: PMC6382501
10. Mawardi H, Alsubhi A, Salem N, Alhadlaq E, Dakhil S, Zahran M, Elbadawi L. Management of medication-induced gingival hyperplasia: a systematic review. Oral Surg Oral Med Oral Pathol Oral Radiol. 2021 Jan;131(1):62-72. doi: 10.1016/j.oooo.2020.10.020. Epub 2020 Oct26. PMID: 33214091.
11. Katsanos KH, Torres J, Roda G, Brygo A, Delaporte E, Colombel JF. Review article: non-malignant oral manifestations in inflammatory bowel diseases. Aliment Pharmacol Ther. 2015 Jul;42(1):40-60. doi: 10.1111/apt.13217. Epub 2015 Apr 28. PMID: 25917394
12. Malins TJ, Wilson A, Ward-Booth RP. Recurrent buccal space abscesses: a complication of Crohn's disease. Oral Surg Oral Med Oral Pathol. 1991 Jul;72(1):19-21. doi: 10.1016/0030-4220(91)90182-c. PMID: 1891238.
13. Femiano F, Lanza A, Buonaiuto C, Perillo L, Dell'Ermo A, Cirillo N. Pyostomatitis vegetans: a review of the literature. Med Oral Patol Oral Cir Bucal. 2009 Mar 1;14(3):E114-7. PMID: 19242389.
14. Pittock S, Drumm B, Fleming P, McDermott M, Imrie C, Flint S, Bourke B. The oral cavity in Crohn's disease. J Pediatr. 2001 May;138(5):767-71. doi: 10.1067/mpd.2001.113008. PMID: 11343060.
15. Harty S, Fleming P, Rowland M, Crushell E, McDermott M, Drumm B, Bourke B. A prospective study of the oral manifestations of Crohn's disease. Clin Gastroenterol Hepatol. 2005 Sep;3(9):886-91. doi: 10.1016/s1542-3565(05)00424-6. PMID: 16234026.
16. Jang HJ, Kang B, Choe BH. The difference in extraintestinal manifestations of inflammatory bowel disease for children and adults. Transl Pediatr. 2019 Jan;8(1):4-15. doi: 10.21037/tp.2019.01.06. PMID: 30881893; PMCID: PMC6382501.
17. Long D, Wang C, Huang Y, Mao C, Xu Y, Zhu Y. Changing epidemiology of inflammatory bowel disease in children and adolescents. Int J Colorectal Dis. 2024 May 18;39(1):73. doi: 10.1007/s00384-024-04640-9. PMID: 38760622; PMCID: PMC11101569.

18. Katsanos KH, Roda G, Brygo A, Delaporte E, Colombel JF. Oral Cancer and Oral Precancerous Lesions in Inflammatory Bowel Diseases: A Systematic Review. J Crohns Colitis. 2015 Nov;9(11):1043-52. doi: 10.1093/ecco-jcc/jjv122. Epub 2015 Jul 10. PMID: 26163301.
19. Lourenço SV, Hussein TP, Bologna SB, Sipahi AM, Nico MM. Oral manifestations of inflammatory bowel disease: a review based on the observation of six cases. J Eur AcadDermatol Venereol. 2010 Feb;24(2):204-7. doi: 10.1111/j.1468-3083.2009.03304.x. Epub 2009 Jun 22. PMID: 19552719.
20. Conrad MA, Bittinger K, Ren Y, Kachelries K, Vales J, Li H, Wu GD, Bushman FD, Devoto M, Baldassano RN, Kelsen JR. The intestinal microbiome of inflammatory bowel disease across the pediatric age range. Gut Microbes. 2024 Jan-Dec;16(1):2317932. doi: 10.1080/19490976.2024.2317932. Epub 2024 Feb 25. PMID: 38404111; PMCID: PMC10900269.
21. Zbar AP, Ben-Horin S, Beer-Gabel M, Eliakim R. Oral Crohn's disease: is it a separable disease from orofacial granulomatosis? A review. J Crohns Colitis. 2012 Mar;6(2):135-42. doi: 10.1016/j.crohns.2011.07.001. Epub 2011 Aug 9. PMID: 22325167.
22. Grave B, McCullough M, Wiesenfeld D. Orofacial granulomatosis--a 20-year review. Oral Dis. 2009 Jan;15(1):46-51. doi: 10.1111/j.1601-0825.2008.01500.x. PMID: 19076470.
23. Veloso FT. Extraintestinal manifestations of inflammatory bowel disease: do they influence treatment and outcome? World J Gastroenterol. 2011 Jun 14;17(22):2702-7. doi: 10.3748/wjg.v17.i22.2702. PMID: 21734777; PMCID: PMC3122258.
